# Knowledge, Attitude, and Practice Regarding High Salt Intake and Association With Hypertension Among Rural Women in Chandpur District of Bangladesh

**DOI:** 10.1002/hsr2.70387

**Published:** 2025-01-23

**Authors:** Pradip Chandra, Aminul Islam, Shahara Robertson, Abu‐Al‐Rafe, Md. Abu Sayed, Afra Anjum Ahona, Mst. Nadira Parvin, Pradip Sen Gupta

**Affiliations:** ^1^ Department of Public Health North South University Dhaka Bangladesh; ^2^ Department of Epidemiology and Biostatistics Bangladesh University of Health Sciences Dhaka Bangladesh; ^3^ Department of Health informatics Bangladesh University of Health Sciences Dhaka Bangladesh; ^4^ Department of Public Health & Informatics Bangladesh University of Professionals Dhaka Bangladesh; ^5^ Department of Epidemiology Bangladesh University of Health Sciences Dhaka Bangladesh

**Keywords:** Bangladesh, high salt intake, hypertension, KAP, rural community, women

## Abstract

**Background and Aims:**

The human body requires a relatively little quantity of sodium to transmit nerve impulses, contract and relax muscles, and maintain appropriate water and mineral balance and which is typically added from diets. The study aimed to assess the level of knowledge, attitude, and practice regarding high salt intake and their association with hypertension among rural women of a selected community in Chandpur.

**Methods:**

A cross‐sectional study was adopted to collect data from 250 households of Chandpur district. The study was conducted during May 2022 to July 2022. The semi‐structured pretested WHO‐modified step‐wise approach to the non‐communicable disease risk factor surveillance (STEPS). Instrument version 3.1 module was used to administer the knowledge, attitude and practice questionnaire to assess the knowledge, attitude and practice of the participants. Binary logistic regression was performed to examine the factor related with knowledge, attitude, and practices.

**Results:**

The result gives evidence that participant who added always salt to their meals were three times more likely to be hypotensive (AOR: 2.99; CI: 1.40, 6.42). Besides, participants who avoid eating outside are significantly less likely (AOR: 0.19; C.I: 0.05 to 0.70) to be hypertensive.

**Conclusion:**

Findings showed that daily salt consumption and “avoid eating out” were significantly associated with hypertension status. These findings imply that further educational intervention is needed to increase understanding of healthy dietary salt intake among rural women.

## 1 Background

Salt or sodium chloride is essential for the proper functioning of the human body [1]. A relatively small amount of sodium is necessary to transmit nerve impulses, contract and relax muscles, and maintain appropriate water and mineral balance which is typically added to diets [2]. The body requires approximately 3.8 g of dietary salt daily. However, excessive salt consumption can have adverse health effects [3, 4]. High salt consumption is a significant contributor to the development of hypertension and other non‐communicable diseases (NCDs) [5], particularly heart disease and stroke [6]. In 2019, NCDs were responsible for approximately 17 million premature deaths (deaths before age 70) with cardiovascular diseases accounting for 38% of these deaths [7].

Salt reduction is among the most cost‐effective public health strategies, recognized by the World Health Organization (WHO) as a “best‐buy” approach [8, 9]. The incidence of hypertension increases with age, impacting over 60% of individuals over 60 years and above [10]. However, adults who consume fewer than 5 g of salt per day had lower blood pressure and a lower risk of heart attack, stroke, and other cardiovascular diseases [11]. Since 2006, WHO has aimed to reduce the global salt intake to fewer than 5 g daily [12]. However, global estimates suggest that adults currently consume about 10 g of salt per day, which is double the WHO recommendation [13]. During the 2013 World Health Assembly, WHO member states were striving to achieve a 30% reduction in salt consumption worldwide by 2025 [14].

A systematic meta‐analysis conducted by members of the South Asian Association for Regional Cooperation (SAARC) countries reported that the average prevalence of hypertension and prehypertension was 27% and 29.6%, respectively [15]. Cardiovascular diseases account for 25% of all deaths (3.6 million), with 8.5 million deaths from NCDs occurring annually in the Southeast Asian region, with 50% of these fatalities being premature [16]. Besides, the traditional dietary habits are likely high in salt, exacerbated by the growing commercialization of processed foods and fast food [17]. In most Southeast Asian countries, adult salt consumption can reach 12 g per day [16].

The “Noncommunicable Diseases: Progress Monitor 2022” report indicates that NCDs account for 70% of all deaths in Bangladesh, which illustrates the total number (557,200) deaths. Additionally, the premature mortality rate due to NCDs is 19% [18]. In Bangladesh, 21% of the adult population has elevated blood pressure and adults consume an average of 9 grams of salt per day [19].

The Bangladesh Health and Demographic Survey 2022 reveals that hypertension is more prevalent in women (23%) compared to men (17%). Among women, the prevalence is 8% among young women aged between 18 and 34 years and 36% among 35 and over. Notably, nearly half of hypertensive women are unaware of their high blood pressure (43%) [20]. However, the relationship between KAPs regarding salt intake and hypertension has never been investigated in this country. Therefore, this study aimed to assess the level of knowledge, attitude, and practice regarding high salt intake and its association with hypertension among rural women in Chandpur.

## 2 Methods and Materials

A cross‐sectional study was conducted in 2022 at Sankibhanga village in Jahirabad union to assess the salt consumption pattern of the rural women of Chandpur. The knowledge, attitudes, and practice (KAP) were assessed through face‐to‐face interviews using a structured questionnaire. The study was ethically approved by the Bangladesh University of Health Sciences Ethical Review Committee (BUHS/ERC/EA/22/303) and written consent was obtained from all participants prior to the interview. Data collection permission was secured from the chairman of the Union.

### 2.1 Study Participants and Sampling

Participants of this study consisted of women aged 18 years and above from Sankibhanga. The Jahirabad Union was selected purposively. That union comprises eight villages and Sankibhanga was chosen by lottery. A convenience sample technique was employed to select the 250 participants and the eligible household member present at home during the interview was invited to take part in the survey. One participant was chosen for the interview from each home and the data were collected from the selected community.

### 2.2 Data Collection Procedures

Survey responses were collected verbally and documented on paper. Demographic data collected included age, religion, marital status, employment, education, family size, and monthly income. The assessment of KAP was conducted using the WHO‐modified step‐wise approach to the NCD risk factor surveillance (STEPS) Instrument version 3.1 module for salt estimation. This included four questions on knowledge and attitudes to assess understanding of the relationship between high salt intake and health complications and the recommended intake level, perception of salt intake, and the importance of reducing salt in diet. A total of 10 questions were posed to measure the frequency of salt‐related practice. Three of these questions were rated on a 5‐point Likert scale that focused on adding more salt to meals or at the table, consuming processed foods high in salt, and preparing meals with salt. Additionally, seven questions with binary responses assessed salt consumption practices such as avoiding processed foods and eating outside.

For clinical measurements, such as the body mass index (BMI), which is determined by taking their height, weight, and blood pressure history are recorded simultaneously with the patient's report. Height was measured by the height measurement tape while weight was measured using a weight machine. Additionally, self‐reported blood pressure history was measured through two questions, confirmed by health workers or physicians. Hypertensive was defined as blood pressure exceeding 140/90 mmHg, whereas normotensive was considered within the normal range of 120/80 mmHg [21].

### 2.3 Statistical Analysis

Following data collection, data were entered into an Excel spreadsheet with quality was evaluated whether any missing values and particular response validity. The primary objective was to assess the KAP regarding salt intake across various demographic groups. For categorical and quantitative variables, descriptive (frequency, percentage, mean, and standard deviation) analysis was performed as required. The chi‐square (Fisher's exact test) test with a 5% level of significance was used to compare the categories. Binary logistic regression was performed to determine the association between KAPs and hypertension status. The KAP variables were included according to the variable inflation factor (VIF) and tolerance. VIF values ranged from 1.140 to 1.787 (all below 2), and tolerance values ranged from 0.56 to 0.88 (all above 0.2), indicating no multicollinearity concerns. The significance level was set at 5% for binary logistic regression. Statistical analysis was conducted using IBM SPSS Version 26.

## 3 Results

### 3.1 Demographic Characteristics

The survey included 250 female participants with a mean age of 40 years ± (SD 12 years). Table 1 shows that most of the women (43.6%) were aged between 18 and 35 years. Among them, the majority of the participants (84.4%) were Muslim, and 74% had completed elementary education (up to the primary level). Most of the participants (98.4%) were married and 68.8% had 1–5 family members. Sixty‐two percent of participants had a monthly household income ranging from 10,000 to 20,000 Taka. Over half of them (53.2%) had a healthy BMI, while 28.8% did not. Approximately 58% of women had heard about the consequences of high salt intake from a physician or health worker whereas 26.4% had never heard such information. During the study period, 82% of the participants did not exhibit elevated blood pressure or hypertension.

**Table 1:** Demographic distribution of the participants (*n* = 250).

Variables		Frequency	Percentage (%)
Age	18–35 Years	109	43.6
	36–50 Years	83	33.2
	> 50 Years	58	23.2
Religion	Muslim	211	84.4
	Hindu	39	15.6
Educational status	Up to primary a	185	74
	Secondary and above b	65	26
Marital status	Married	246	98.4
	Unmarried	4	1.6
Family member	1–5 Members	172	68.8
	More than 5 members	78	31.2
Monthly income	< 10,000 Tk	29	11.6
	10,000–20,000 Tk	155	62
	> 20,000 Tk	66	26.4
Body mass index	Underweight	19	7.6
	Healthy weight	133	53.2
	Overweight	72	28.8
	Obese	26	10.4
Source of information	Physician or Health worker	144	57.6
	Other sources c	40	16
	Never heard	66	26.4
Hypertension	Yes	45	18
	No	205	82

^a^ Up to primary: No formal education, primary.

^b^ Secondary and above included: Secondary, higher secondary, postgraduate and over.

^c^ Other sources included: Newspapers, television, and social media.

### 3.2 Knowledges and Attitudes Regarding Dietary Salt Consumption

Table 2 summarizes the distribution of knowledge and attitude among the participants. Thirty‐eight percent of respondents reported consuming excessive salt, while 38.4% believed they were consuming the appropriate amount of salt. Over half of the participants (52%) considered reducing salt intake to be very important. Additionally, 77.2% of respondents acknowledged that a high salt diet could lead to major health issues. However, the majority of participants (69.2%) were unaware of the recommended daily salt allowance, and only 12% had prior knowledge regarding the appropriate amount of salt consumption.

**Table 2:** Knowledge and attitudes regarding dietary salt consumption of the participants (*n* = 250).

Variable		Frequency	Percentage (%)
Knowledge of the recommended daily intake of salt	Less than 5 g (1 teaspoon)	30	12
	Less than 2 g (1/2 teaspoon)	47	18.8
	Don't know	173	69.2
Higher salt in the diet can cause serious health problem	Yes	193	77.2
	No	57	22.8
The significance of reducing salt intake	Very important	130	52
	Somewhat important	81	32.4
	Not at all important	37	14.8
	Don't know	2	0.8
Perception of salt intake	Far too much	39	15.6
	Too much	95	38
	Just the right amount	96	38.4
	Too little	17	6.8
	Don‎'t know	3	1.2

### 3.3 Practice Regarding Salt Intake

Table 3 highlights the practices related to salt intake among the participants. Nearly two‐thirds of the women reported “always added salt” during their meals. All respondents added salt during preparing foods in their household. Approximately 62% of participants occasionally consumed processed foods, such as chutneys and pickles which are high in salt. Additionally, 77.2% of participants stated that they avoid processed foods. None of the women checked the salt content of the product and never purchased low‐sodium salts. They consistently prepared their meals with added salt and spices. Moreover, about 62% of the participants reported that they avoided eating outside.

**Table 3:** Regular practices regarding dietary salt consumption of the participants (*n* = 250).

Variables		Frequency	Percent (%)
Added salt during meal	Always	183	73.2
	Sometimes	15	6
	Rarely/Never	52	20.8
Adding salt while preparing or cooking meals at home	Always	250	100
How frequently do you consume processed foods like chutneys and pickles that are high in salt?	Always	4	1.6
	Sometimes	154	61.6
	Rarely	81	32.4
	Never	11	4.4
Avoid eating processed foods	Yes	193	77.2
	No	57	22.8
Look at the amount of salt in the food	No	250	100
Consume food without adding salt to the table	Yes	50	20
	No	200	80
Buy low‐sodium salts	No	250	100
Prepared food without using salt	No	250	100
When cooking, use spices instead of salt.	Yes	250	100
Avoid eating out	Yes	154	61.6
	No	96	38.4

### 3.4 KAP Association With Hypertension Status

Table 4 present the hypertension status across various KAP variables. It highlighted that added salt during meal, frequent consumption of processed foods, perceived salt consumption, importance of lowering salt in the diet, perception on consuming a diet high in salt can cause serious health problems, perceived appropriate salt consumption level, consumption of food without adding salt on the table, and avoid eating out showing the association between hypertension.

**Table 4:** KAP association among educational status and hypertension status of the participants (*n* = 250).

KAP		Hypertension status		
		Hypertensive	Normotensive	*p*‐Value
Perception on consuming a diet high in salt can cause serious health problems (*n* = 250)	Yes	41(91.1%)	152(74.1%)	0.02
	No	4(8.9%)	53(25.9%)	
Perceived appropriate salt consumption level (*n* = 250)	Less than 5 g (1 teaspoon)	4(8.9%)	26(12.7%)	0.01
	Less than 2 g (1/2 teaspoon)	16(35.6%)	31(15.1%)	
	Don't know	25(55.6%)	148(72.2%)	
Perceived salt consumption (*n* = 247)	Far too much	4(9.3%)	35(17.2%)	0.002
	Too much	9(20.9%)	86(42.2%)	
	Just the right amount	23(53.5%)	73(35.8%)	
	Too little	7(16.3%)	10(4.9%)	
Important of lowering salt in the diet (*n* = 248)	Very important	33(73.3%)	97(47.8%)	0.005
	Somewhat important	10(22.2%)	71(35%)	
	Not at all important	2(4.4%)	35(17.2%)	
Added salt during meal (*n* = 250)	Always	23(51.1%)	160(78%)	< 0.001
	Sometimes	2(4.4%)	13(6.3%)	
	Rarely/Never	20(44.4%)	32(15.6%)	
Frequent consume processed foods (*n* = 250)	Always	0	4(2%)	< 0.001
	Sometimes	20(44.4%)	134(65.4%)	
	Rarely	18(40%)	63(30.7%)	
	Never	7(15.6%)	4(2%)	
Avoid eating of processed foods (*n* = 250)	Yes	39(86.7%)	154(75.1%)	0.12
	No	6(13.3%)	51(24.9%)	
Consume food without adding salt on the table (*n* = 250)	Yes	20(44.4%)	30(14.6%)	< 0.001
	No	25(55.6%)	175(85.4%)	
Avoid eating out (*n* = 250)	Yes	38(84.4%)	116(56.6%)	< 0.001
	No	7(15.6%)	89(43.4%)	

### 3.5 Association of KAP With Hypertension Status

Table 5 illustrates the association between KAP and hypertension status. The participants who always added salt during their meals were about three times more likely to be hypertensive (AOR: 2.99; CI: 1.40, 6.42) compared to those who never or rarely added salt and this result is statistically significant. Furthermore, those who avoided eating outside were significantly less likely to be hypertensive (AOR: 0.19; C.I: 0.05 to 0.70). This result indicates a strong association between avoiding eating out and a lower risk of hypertension.

**Table 5:** Association of KAP with hypertension status and educational status (*n* = 247).

KAP	Hypertension status (Hypertensive, Normotensive; ref)			
	COR (95% CI)	*p*‐Value	AOR (95% CI)	*p*‐Value
Knowledge about the recommended amount of daily salt consumption				
Less than 5 g (1 teaspoon)	1.09 (0.35, 3.42)	0.872	1.95 (0.58, 6.61)	0.28
Less than 2 g (1/2 teaspoon)	0.33 (0.16, 0.69)	0.003	0.50 (0.22, 1.13)	0.10
Don't know	Ref		Ref	
Too much salt in diet could cause a serious health problem				
Yes	0.28 (0.10, 0.82)	0.02	0.35 (0.11, 1.08)	0.07
No	Ref		Ref	
Added salt during meal				
Always	4.348 (2.14, 8.84)	< 0.001	2.99 **(**1.40, 6.42)	**0.005**
Sometimes	4.06 (0.83, 19.92)	0.084	2.22 (0.41, 12.11)	0.36
Rarely/Never	Ref		Ref	
Avoid consumption of processed foods				
Yes	0.47 (0.19, 1.16)	0.101	1.98 (0.48, 8.24)	0.35
No	Ref		Ref	
Avoid eating out				
Yes	0.24 (0.1, 0.56)	0.001	0.19 (0.05, 0.69)	**0.012**
No	Ref		Ref	

Abbreviations: AOR: adjusted odds ratio; CI: confidence interval; COR: crude odds ratio.

## 4 Discussion

To the best of our knowledge, this study is the first to explore the KAP regarding salt intake in association with hypertension in Bangladesh. The findings reveal a significant association between hypertension and eating outside and adding salt every day. A substantial gap in the existing literatures is the limited focus on KAP concerning hypertension status. This study focused on this potential research gap that needs to be addressed. Due to the scarcity of similar studies, there was a limited amount of literature available for comparison.

### 4.1 Knowledge

This study demonstrated that 77.2% had good knowledge regarding the health risks associated with high salt intake in the diet, a rate higher than the general Bangladeshi population's 60.7%, another domestic study was conducted among university faculty members and doctors showed an even higher proportion 93.5%, which might be due to their professional and educational backgrounds [22, 23]

This study finds that knowledge of the recommended amount of daily salt consumption had no significant association with hypertension. Additionally, there was no significant relation between hypertension and women who were aware that a high salt in diet could cause a serious health problem, contrasting with the findings from a study conducted on the Malaysian population [24]. These findings suggest that while participants had good knowledge, their actual practice regarding salt reduction was lacking. Therefore, more awareness programs could be beneficial to enhancing both their understanding and implementation of salt reduction practices.

### 4.2 Attitude

A study reported that 52% of respondents considered reducing salt intake to be very important, which was higher than the Bangladeshi population's view of 38.8%. However, this was remarkably lower than that of faculty and doctors (72.8%), undergraduate medical students (54.4%), and Chinese adults (85.2%) [1, 13, 25]. This study also revealed that nearly 38.4% of participants believed they consume the right amount of salt, which is higher than the university faculty/doctor participants (29.3%) [13]. These attitudes reflect an overestimation, a possible reason could be the limited knowledge of recommended salt consumption. An educational awareness campaign could be an effective intervention to provide suitable guidelines regarding salt intake.

### 4.3 Practices

The study result indicated that women who consistently added salt during their meals were three times more likely to be hypertensive compare to those who rarely or never added salt. This finding is supported by a similar study conducted in Iran, which found that hypertensive participants were about three times more likely to consume salt during their meals. This finding indicates that many participants may be unaware of their salt consumption [26]. Implementing a community‐based awareness program could enhance their knowledge and practice on salt reduction. Another interesting finding revealed that participants who avoided eating out were 80% less likely to be hypertensive compared to those who ate outside. This finding contrasts with the Malay Elderly population study, which showed no significant association between this practice and blood pressure [27].

This study explored that 76.8% of the participants avoided processed foods, a significantly higher proportion compared to the general population (46.8%) of Bangladesh and also higher than another study conducted among nurses (64.5%) [28, 29]. It was also found that none of the participants checked the salt levels in foods contrasting with a study where 33.7% of university faculty and doctors did so. Additionally, none of the participants in this study typically purchased low‐sodium salt, whereas the university faculty members and doctors reported using low‐salt and sodium alternatives, with about 41% of nurses also opting for low‐sodium salt [13, 28].

### 4.4 Strengths and Limitations

This study can be used as a baseline for monitoring modifications in practice, marking the first to evaluate KAPs associated with hypertension in Chandpur. However, the study had some drawbacks. Initially, there was little chance of information bias due to self‐reported responses. Secondly, the study's small sample size was influenced by the rural women's unfamiliarity with the survey and specific social barriers. Finally, as the study was conducted in a small community in Chandpur, the findings may not entirely represent all rural women of Bangladesh.

## 5 Conclusion

Knowledge did not show a significant relation with hypertension. However, the practice of daily salt consumption and “avoid eating out” were significantly associated with hypertension status. As part of the prevention of hypertension, these findings imply that further educational interventions are needed to increase understanding of healthy dietary salt intake among rural women. The results suggest the population‐level salt reduction initiatives. Moreover, further research is necessary to comprehend the outcomes.

## Author Contributions

**Pradip Chandra:** designed the work, processed the data, developed the methodology, wrote the first draft, reviewed it and wrote the manuscript. **Aminul Islam:** ideation, management of the study, verification, and manuscript composition. **Shahara Robertson:** methodology, data curation, and subsequent manuscript modification. **Abu‐Al‐Rafe:** manuscript drafting, review, study management, and data curation. **Md. Abu Sayed:** formal analysis, ideation, illustration, and ensuing manuscript modification. **Afra Anjum Ahona:** original draft writing, methodology, data curation, and visualization. **Mst. Nadira Parvin:** ideation, verification, research, and ensuing paper modification. **Pradip Sen Gupta:** formal analysis, methodology, conception, validation, oversight, follow‐up revision, and original article writing. The authors all concur to take responsibility for all parts of the work and have approved the final text as submitted.

## Ethics Statement

This study was ethically approved by the Bangladesh University of Health Sciences Ethical Review Committee (BUHS/ERC/EA/22/303).

## Consent

The study participants provided written consent before the interview. Data collection permission was taken from the chairman of that Union.

## Conflicts of Interest

The authors declare no conflicts of interest.

### 1 Transparency Statements

The lead author Pradip Chandra affirms that this manuscript is an honest, accurate, and transparent account of the study being reported; that no important aspects of the study have been omitted; and that any discrepancies from the study as planned (and, if relevant, registered) have been explained.

## Data Availability

The data that support the findings of this study are available from the corresponding author upon reasonable request. The corresponding author may provide the data support upon reasonable request. All author has access to the data of this article. Any researcher who has an appropriate reason and interest they may contact with the corresponding author (Pradip Chandra‐ pradipchandra@buhs.ac.bd).
